# Dopamine Neuron Stimulating Actions of a GDNF Propeptide

**DOI:** 10.1371/journal.pone.0009752

**Published:** 2010-03-18

**Authors:** Luke H. Bradley, Josh Fuqua, April Richardson, Jadwiga Turchan-Cholewo, Yi Ai, Kristen A. Kelps, John D. Glass, Xiuquan He, Zhiming Zhang, Richard Grondin, O. Meagan Littrell, Peter Huettl, Francois Pomerleau, Don M. Gash, Greg A. Gerhardt

**Affiliations:** 1 Department of Anatomy & Neurobiology and the Morris K. Udall Parkinson's Disease Research Center of Excellence, University of Kentucky College of Medicine, Lexington, Kentucky, United States of America; 2 Department of Molecular & Cellular Biochemistry and the Center of Structural Biology, University of Kentucky College of Medicine, Lexington, Kentucky, United States of America; 3 Shoreham, New York, United States of America; University of Nebraska, United States of America

## Abstract

**Background:**

Neurotrophic factors, such as glial cell line-derived neurotrophic factor (GDNF), have shown great promise for protection and restoration of damaged or dying dopamine neurons in animal models and in some Parkinson's disease (PD) clinical trials. However, the delivery of neurotrophic factors to the brain is difficult due to their large size and poor bio-distribution. In addition, developing more efficacious trophic factors is hampered by the difficulty of synthesis and structural modification. Small molecules with neurotrophic actions that are easy to synthesize and modify to improve bioavailability are needed.

**Methods and Findings:**

Here we present the neurobiological actions of dopamine neuron stimulating peptide-11 (DNSP-11), an 11-mer peptide from the proGDNF domain. *In vitro*, DNSP-11 supports the survival of fetal mesencephalic neurons, increasing both the number of surviving cells and neuritic outgrowth. In MN9D cells, DNSP-11 protects against dopaminergic neurotoxin 6-hydroxydopamine (6-OHDA)-induced cell death, significantly decreasing TUNEL-positive cells and levels of caspase-3 activity. *In vivo*, a single injection of DNSP-11 into the normal adult rat substantia nigra is taken up rapidly into neurons and increases resting levels of dopamine and its metabolites for up to 28 days. Of particular note, DNSP-11 significantly improves apomorphine-induced rotational behavior, and increases dopamine and dopamine metabolite tissue levels in the substantia nigra in a rat model of PD. Unlike GDNF, DNSP-11 was found to block staurosporine- and gramicidin-induced cytotoxicity in nutrient-deprived dopaminergic B65 cells, and its neuroprotective effects included preventing the release of cytochrome c from mitochondria.

**Conclusions:**

Collectively, these data support that DNSP-11 exhibits potent neurotrophic actions analogous to GDNF, making it a viable candidate for a PD therapeutic. However, it likely signals through pathways that do not directly involve the GFRα1 receptor.

## Introduction

A hallmark of Parkinson's disease (PD) is the degeneration of dopamine neurons in the pars compacta of the substantia nigra [1]. Glial cell line-derived neurotrophic factor (GDNF), has been extensively shown to be a promising PD therapeutic that promotes the survival of dopamine neurons, protects against neurotoxin-induced injury, and has powerful restorative effects for damaged or dying dopamine neurons [2]–[8]. However, the widespread clinical application of GDNF has been hindered, in part, as a consequence of its heparin-binding domains and large molecular size limiting brain diffusion [6]. Thus, finding a small molecule with potent neurotrophic effects has important implications in the treatment of PD.

The emergence of naturally occurring, physiologically functional propeptides from the neurotrophic factor family provides a wealth of untapped sequences for exploration and pharmacological evaluation [9]. Neurotrophic factor propeptide sequences have historically been thought to have very little - if any - function, except to promote protein folding and regulation of secretion [10], [11]. However, the prodomains of nerve growth factor (NGF) and brain derived neurotrophic factor (BDNF) have been determined to bind to the pro-apoptotic receptor, sortilin, and promote cell-death by recruiting the mature p75NTR receptor when the dibasic furin cleavage site has been mutated (removed) between the prodomain and the mature growth factor [12]–[14]. In addition, studies have shown that the isolated prosequence of NGF can be used to block the induction of apoptosis, by likely preventing the proapoptotic ternary complex (sortilin-p75NTR-proNGF) formation [13]. While these findings are quite surprising, they strongly suggest that the highly conserved prosequences of other related neurotrophic factors contain novel, physiologically relevant functions.

**Figure 1:**
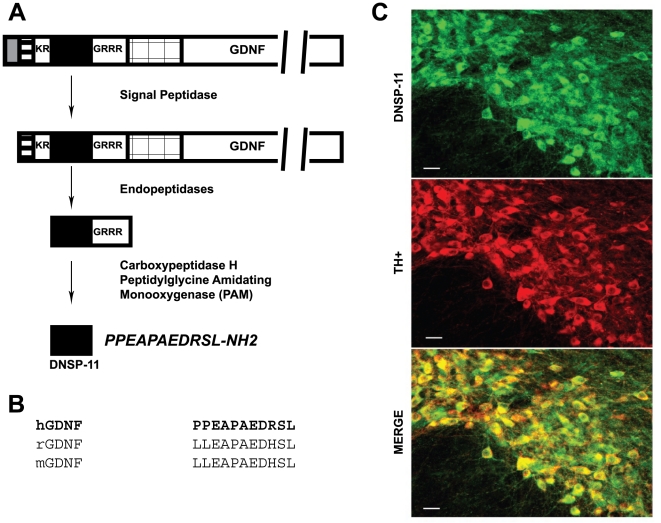
Sequence origin and homology of DNSP-11. (A) DNSP-11 (filled) is an 11 amino acid sequence present in the proprotein region of the 211 amino acid human pre-proGDNF sequence. After cleavage of the pre-signal sequence (gray), DNSP-11 is predicted to be cleaved from the proprotein at flanking dibasic cleavage sites by endopeptidases. Further predicted processing yields the C-terminal amidated peptide. The N-terminal (striped) and C-terminal (checkered) proprotein fragments and mature GDNF (open) protein are shown. The sequence figure is not drawn to scale to highlight the processing of DNSP-11. (B) DNSP-11 shows high sequence homology to the rat and mouse proGDNF sequences suggesting a conserved function. (C) *In vivo* expression of the DNSP-11 sequence in the substantia nigra of the ventral mesencephalon from rat pups at PN10. Rows indicate the type of stain: the top panel is DNSP-11 (green); the middle panel is a dopaminergic neuron marker, tyrosine hydroxylase (TH^+^, red); the bottom panel represents a merged image of the previous stains (yellow). The bottom panel demonstrates co-localization of DNSP-11 sequence within dopaminergic cell bodies at PN10. The scale bar represents 30 µm.

Like all neurotrophic factors, GDNF is endogenously produced as a proprotein following signal peptidase cleavage of the N-terminal 19 amino acid residue presequence [2]. Examination of the human GDNF prosequence predicts internal dibasic endopeptidase sites that would yield an 11-mer amidated peptide named Dopamine Neuron Stimulating Peptide-11 (DNSP-11), upon proteolytic processing (**Figure 1A**) [15]. Independently, the rat homolog of DNSP-11, named brain excitatory peptide (BEP), was found to be a functional neuropeptide, exhibiting an increase in synaptic excitability [15]. Here we present that DNSP-11 exhibits potent neurotrophic actions analogous to mature GDNF, making it a viable candidate for a PD therapeutic, but it likely signals through pathways that do not directly involve the GFRα1 receptor. These data strongly support that the pro-peptides of GDNF, and other neurotrophic factors, may have novel, long-overlooked physiological and potentially therapeutic functions.

## Methods

### Ethics Statement

All animal procedures were approved by our Institutional Animal Care and Use Committee following AAALACI guidelines.

### Materials

Unless otherwise stated, all cell reagents and assays were purchased from Invitrogen. All other materials and chemicals are reagent grade. B65 cells were obtained from ECACC. The polyclonal rabbit anti-hDNSP-11 antibody was produced by Alpha Diagnostic (San Antonio, Texas).

### DNSP-11 and Biotinylated DNSP-11

DNSP-11 (sequence: PPEAPAEDRSL-amide) and biotinylated DNSP-11 (bDNSP-11; sequence: biotin-PPEAPAEDRSL-amide) were synthesized and RP-HPLC purified to >98% by AC Scientific (Duluth, GA) and the W.M. Keck Foundation Biotechnology Resource Laboratory at Yale University. Peptides were characterized for purity and correct sequence by MALDI-TOF LC-MS and Edman degradation. DNSP-11 was determined to be stable, *in vitro*, at a variety of experimentally relevant concentrations and temperatures, including 37°C in sterile pH 5 citrate buffer for 31 days.

### Tissue preparation for DNSP-11 Staining in Substantia Nigra at Postnatal Day 10 (PN10)

Tissue was prepared from Sprague Dawley (SD) pups. Brains were rinsed in Dulbecco's Phosphate Buffered Saline (DPBS, Gibco), and submerged in 4% paraformaldehyde pH 7.4 for 48 hours. Following submersion in 30% sucrose, brains were sectioned coronally (40 µm) and stored in cryoprotectant solution at −70°C until processed for immunohistochemistry.

### DNSP-11 treatment of Mesencephalic Cells

Timed pregnant SD rats (Harlan) were used to obtain the ventral mesencephalon from E14 fetuses. The dissected tissue was collected in cold Neurobasal™ medium and rinsed twice with cold PBS. The cells were chemically (TrypLE®) and mechanically dissociated to yield a single cell suspension. The solution was centrifuged at 169 g for 6 minutes and the pellet re-suspended in Dulbecco's Modified Eagle Medium (DMEM). Cells were plated in a 25 µL micro-island at a density of 4000 cells/ µL on poly-D-lysine coated 24-well plates (Sigma). Following adherence, cells were supplemented with warm Neurobasal™ media containing 2 mM glutamine and 100 units of penicillin/streptomycin. Neurotrophic compounds were added at each media addition, including initial plating and DIV 2. Peptides (0.03 ng to 10 ng/mL) were added to a 24-well plate following media supplementation.

### MN9D and B65 Cell Cultures

MN9D [16] and B65 [17] cells were cultured in DMEM supplemented with 10% Fetal Bovine Serum (FBS, Hyclone), 50 U/mL penicillin and streptomycin. For experiments, the cells were plated on 24-well poly-D-lysine in DMEM with 1% (v/v) penicillin-streptomycin. The cells were grown at 37°C in 5% CO_2_.

### Caspase-3 Activity Assay in MN9D Cells

MN9D cells were plated to 100,000 cells/well. Cell cultures were exposed to DNSP-11 (1 ng/mL) or buffer for 1 hour prior to 15 min 100 µM 6-OHDA exposure. Caspase-3 activity was monitored after 3 hours by fluorescence (λ_ex_/λ_em_ 496/520 nm) using the Enz Chek Caspase-3 kit. Protein levels of lysed cells were measured by BCA assay (BioRad) and normalized for every experiment. Data are expressed as % control and were repeated a minimum of 3 times.

### Terminal dUTP Nick-End Labeling (TUNEL) Assay in MN9D Cells

After treatment with DNSP-11, MN9D cells were fixed and labeled to assess degenerative nuclear changes as indicated by the extent of high-molecular weight DNA strand breaks. DNA fragmentation was detected by using streptavidin-horseradish peroxidise conjugate followed by the substrate diaminobenzidine (DAB) generating a colored precipitate. Ratios between apoptotic and total cells were determined (4 random fields/well; 4 wells/ group). Experiments were repeated 3 times.

### LIVE/DEAD® Calcein AM/ Ethidium homodimer (EthD-1) Assay

B65 cells were cultured in 96-well plates for 24 h and then incubated with 2 µM calcein AM and 4 µM EthD-1 in PBS, at RT for 60 min (LIVE/DEAD® Viability/Cytotoxicity Assay Kit). The hydrolysis of calcein AM in the cytoplasm of live cells was monitored by fluorescence (λ_ex_/λ_em_ 485 nm/530 nm). EthD-1 binding to nucleic acids in damaged cells was monitored by fluorescence (λ_ex_/λ_em_ 530 nm/645 nm). Background fluorescence readings (cell-free control) were subtracted from all values prior to calculation of results. The data were normalized to the fluorescence in vehicle-treated cells and expressed as percent of control ±S.E.M. of three to four independent experiments.

### Cytochrome C Immunostaining

B65 cells were plated on coverslips in 24-well plate. Cells were incubated for 30 min with Mitotracker® Red 0.1 µg/ml to stain mitochondria with red fluorescence. After 30 min the medium was replaced with DMEM medium (no FBS) and treated with staurosporine (1 µM), DNSP-11 (10 ng/ml) and GDNF (1 ng/ml) for 6 h. Each experiment was performed in triplicate wells. The cells were fixed in 4% paraformaldehyde, permeabilized with 0.1% triton-x100, and immunostained with monoclonal antisera to cytochrome C at 1∶1000 dilution. Alexa-488 conjugated goat anti-mouse IgG (1∶500) was used as a secondary antiserum. The coverslips were mounted onto slides with mounting media containing DAPI (VECTOR) to stain the nuclei with blue fluorescence.

### Double Fluorescent Immunostaining of DNSP-11

Floating sections were pretreated with 0.2% H_2_O_2_ in potassium phosphate buffered saline (KPBS) for 10 minutes and blocked with 4% normal goat serum in KPBS for 1 hour. Then, sections were incubated overnight with both rabbit anti-hDNSP-11 polyclonal antibody (1∶2000, Alpha Diagnostic) and mouse anti-TH antibody (1∶1000, Chemicon) in KPBS at 4°C. After washing with KPBS, the sections were incubated with Alexa-488 conjugated goat anti-rabbit IgG (1∶500, Molecular Probes) and Alexa-568 conjugated goat anti-mouse IgG (1∶500, Molecular Probes) for 3 hours. The sections were washed extensively and visualized with a Nikon fluorescence microscope.

### Animals and Surgical Procedures for Normal and 6-OHDA-Lesioned Rats

Fischer 344 rats were used for all experiments and maintained under a 12 hour light/dark cycle with food and water provided *ad libitum*. All animal procedures were approved by our Institutional Animal Care and Use Committee following AAALACI guidelines.

### Infusion Delivery of DNSP-11 or Vehicle

Isoflurane anesthetized (1.5–2.5%) Fischer 344 rats received 5 µl of 6 µg/µL DNSP-11 solution or citrate buffer vehicle solution in a blinded manner. Treatment was delivered to the nigral cell bodies using the same stereotaxic coordinates and protocol for solution delivery as in studies of GDNF [16].

### Reverse Microdialysis

Reverse in vivo microdialysis was accomplished using previously published methods and brain coordinates [18]. CMA 11 microdialysis probes with a 4.0 mm membrane length and 6 kDa molecular weight cut-off were placed within the rat striatum.

### Unilateral 6-OHDA Lesions

The 6-OHDA solution was delivered to two injection sites along the medial forebrain bundle (MFB) using a previously published protocol [19]. Five weeks after the unilateral 6-OHDA MFB lesion procedure, animals were grouped based on apomorphine (0.05 mg/kg, s.c.)-induced rotational behaviour: animals with >300 rotations per 60 minutes were selected. Lesioned animals received 5 µL of either a 20 µg/µL DNSP-11 solution or citrate buffer vehicle solution in a manner similar to infusion delivery in normal animals.

### Neurochemical Content of Tissue

Lesioned animals were euthanized 5 weeks after DNSP-11 or vehicle infusion. The brains were sliced into 1 mm thick sections. Tissue punches were taken from the striatum and the substantia nigra and they were weighed, quick frozen and stored at -70°C until they were assayed by high performance liquid chromatography with electrochemical detection [20].

### Apomorphine–induced Rotational Behavior Testing

Lesion severity was assessed prior to DNSP-11 treatment using apomorphine (0.05 mg/kg, s.c.)- induced rotational behavior. Beginning one week after DNSP-11 treatment, apomorphine-induced rotational behavior was monitored weekly for four weeks [7], [21].

### DNSP-11 Pull-down Assay with Rat Substantia Nigra Homogenate

Fischer 344 rat substantia nigra was homogenized in homogenization buffer (modified from [22] with 20 mM HEPES, pH 7.4) and cytosolic fraction (supernatant) collected after 30 minutes at 100,000 g. 50 µg of bDNSP-11 was incubated with fraction for 15 minutes on ice. Sample was added to streptavidin magnetic beads (New England Biolabs), pelleted, and washed four times in homogenization buffer. Bound proteins were eluted by Solubilization/Rehydration Solution (7 M Urea, 2 M Thiourea, 50 mM DTT, 4% CHAPS, 1% NP-40, 0.2% Carrier ampholytes, 0.0002% Bromophenol blue), and analyzed by 2D-PAGE (BioRad) and later identified by MALDI-TOF MS/MS. MS data were compared to the Uniprot [23] database utilizing the Paragon™ algorithm [24] in ProteinPilot Version 2.0 (Applied Bioscience).

## Results

DNSP-11 is an 11-mer peptide that we and others have predicted to be an endopeptidase cleavage product from the human GDNF prosequence (**Figure 1A**) [15]. Homologous sequences have also been predicted from the rat and mouse GDNF prosequence (**Figure 1B**) [15]. Endogenous immunostaining for DNSP-11 in the mesencephalon of Sprague Dawley (SD) rats indicates that the sequence is uniformly distributed throughout the perikaryal cytoplasm of tyrosine hydroxylase positive (TH+) labeled neurons of the substantia nigra at postnatal day 10 (PN10; **Figure 1C**) analogous to GDNF, while immunostaining in the striatum is at background levels (data not included). In addition, the DNSP-11 sequence has also been detected in the olfactory bulb, granule cells in the hippocampus, granule cells in the cerebellum and the locus coeruleus (data not included). The observed punctate (granular) immunofluorescence comes from labeled neurites emanating from labeled cell bodies. The source of the DNSP-11 seen in nigral dopamine neurons is not known at this time. Immunostaining with this polyclonal antibody does not distinguish between the 11-mer peptide or proGDNF form of the DNSP-11 sequence.

**Figure 2:**
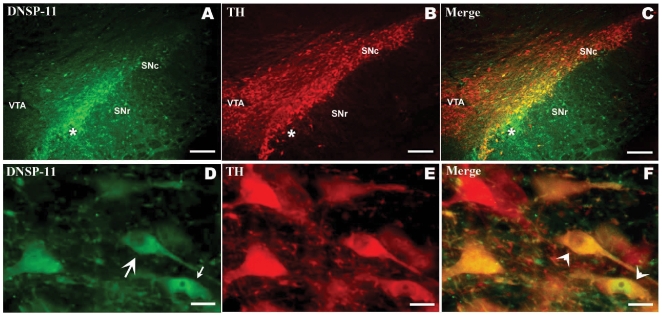
Uptake of DNSP-11 into the rat substantia nigra 30 minutes after a single injection. The fluorescent immunostaining for DNSP-11 (A), TH (B) and the two photomicrographs merged (C) show that DNSP-11 is taken up by neurons in both the substantia nigra, pars reticulata (SNr) and substantia nigra, pars compacta (SNc). TH-positive dopamine neurons (B) populate the SNc and the ventral tegmental area (VTA). Figures D-F are higher power micrographs from the SNc. Immunostaining (D) revealed uptake of DNSP-11 into the perikaryon (large arrow), nucleus (small arrow), and neurites of TH+ cells (E), which appear yellow in the merged imaged (F), including the two cells denoted by arrowheads. The injection site is indicated (star). Scale bar = 200 µm in A-C; 15 µm in D-F.

To determine if DNSP-11 is actively taken up into dopamine-containing neurons *in vivo*, a single administration of 30 µg of DNSP-11 was delivered into the right rat substantia nigra. Animals were euthanized at 0.5, 1.5, 4, 24 and 48 hrs after injection to visualize distribution of DNSP-11 using a polyclonal antibody raised against DNSP-11. As seen in **Figure 2**, DNSP-11 antibodies labeled the cytosol and neurites of neurons in the area of the substantia nigra within 30 minutes after injection. In addition, staining for TH+ and DNSP-11 showed overlap in the pars compacta of the substantia nigra and some DNSP-11 labelling in the pars reticulata - supporting potential uptake into γ-aminobutyric acid-ergic (GABAergic) neurons. The pars reticulata serves as a major efferent pathway from the basal ganglia to the thalamus and brainstem. Thus drugs that affect neurons in the pars reticulata could have significant therapeutic value. Immunohistochemical staining for DNSP-11 diminishes 3 hrs after injection and was absent at 24 hrs and beyond (data not shown), supporting that there is a rapid uptake of DNSP-11 into neurons.

**Figure 3:**
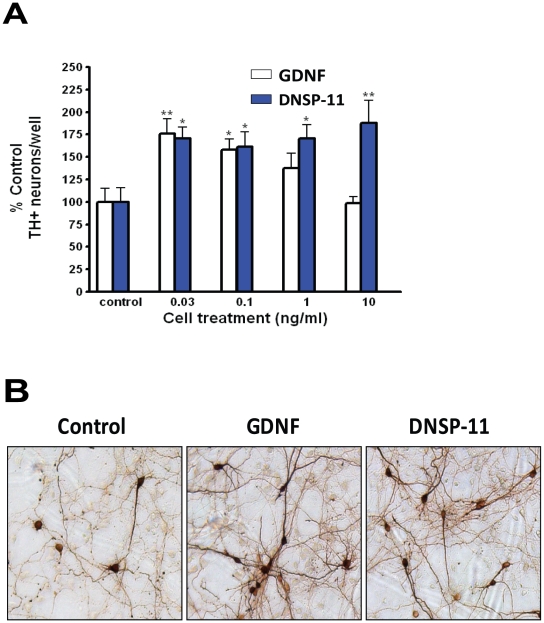
Neurotrophic effects of DNSP-11 and GDNF in Primary Dopaminergic Neurons. (A) E14 rat embryo primary dopaminergic neurons from the ventral mesencephalon were grown for 5 days *in vitro* and neurotrophic molecules were added at each media change, including initial plating and day 2. GDNF (open bars) and DNSP-11 (blue bars) were added at various concentrations (0.03, 0.1, 1.0 and 10 ng/ml; 10 mM citrate buffer +150 mM NaCl, pH 5) and were seen to significantly increase TH+ neuron counts (+ SE; one-way ANOVA with Newman-Keuls post hoc analysis, *p<0.05 and **p<0.01) (B) Photographs of treated E14 primary dopaminergic neurons demonstrating that both GDNF and DNSP-11 treated cells (0.1 ng/ml) displayed enhanced cell survival, neurite length, and total number of branches.

We studied the neurotrophic effects of DNSP-11 by comparing it to the well-known effects of GDNF on the maintenance of primary mesencephalic cell cultures from E14 SD rat embryos. DNSP-11 significantly increased cell survival 75% over citrate buffer control, as indicated by immunocytochemical staining of TH+ neurons 5 days *in vitro* (**Figure 3A**). As previously observed, GDNF produces a decrease in TH+ staining at higher dosages [25], [26]. However, DNSP-11's effects remained constant between 0.03 to 10 ng/mL (Figure 3A). Additional dosing studies are necessary to determine the upper and lower limits of DNSP-11 in primary cell culture. Furthermore, DNSP-11 significantly enhanced morphological changes (**Figure 3B**) consistent with a neurotrophic molecule including: neurite length, total number of branches, and increased total number of TH+ cells (**Table 1**). These effects were similar to those observed for GDNF [2] in these cells, including an increase in the size of TH+ neurons, which was not observed for DNSP-11 (**Table 1**).

**Table 1:** E14 Primary mesencephalic neuron survival and morphological data following treatment with GDNF and DNSP-11.

	GDNF		DNSP-11	
	Control (n)	0.1 ng/mL (n)	Control (n)	0.1 ng/mL (n)
**Cell survival**	100±15 (8)	* 158±12 (8)	100±16 (7)	*161±17 (7)
**Combined neurite length (**µ**m)**	242±12 (135)	** 310±16 (106)	222±11 (139)	** 306±23 (59)
**Soma size (**µ**m^2^)**	171±4 (135)	177±4 (106)	168±3 (139)	165±5 (59)
**Average branches per neuron**	3.8±0.2 (135)	** 4.7±0.2 (106)	3.1±0.2 (139)	** 4.4±0.3 (59)

Cell survival and morphological parameters were quantified for control (citrate buffer) and experimental (0.1 ng/ml GDNF or 0.1 ng/ml DNSP-11) conditions. For morphology, five fields per well (minimum of 15 cells/field; 3–4 independent experiments) were photographed at 20x magnification and quantified using a Bioquant Image Analysis System. DNSP-11 increased cell survival and morphological parameters comparable to GDNF, including combined neurite length and total branches. Soma size was not increased by the addition of DNSP-11. A one-way ANOVA was used to test for significance among groups, followed by a Newman-Keuls post hoc analysis. Significance between control and experimental conditions was determined at *p<0.05 and **p<0.01.

**Figure 4:**
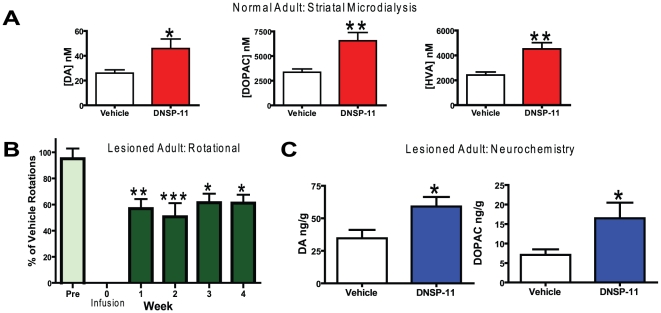
Neurotrophic effects of DNSP-11 *in vivo*. (A) 28 days after DNSP-11 (30 µg) or citrate buffer vehicle was delivered to the nigral cell bodies, neurochemical studies were carried out in the ipsilateral striatum with *in vivo* microdialysis and levels of DA and its metabolites, DOPAC and HVA were determined. The DNSP-11 treatment group showed significantly higher basal neurochemical concentrations of DA, DOPAC and HVA. Basal DA increased from 26.0±2.7 nM in the vehicle treatment group to 45.8±7.7 nM in the DNSP-11 treatment group (t_(31)_ = 2.255, p = 0.0314). Basal concentrations of DOPAC increased from 3355±338 nM in the vehicle group to 6544±836 nM in the DNSP-11 group (t_(31)_ = 3.293, p = 0.0025), and HVA, increased from 2419±251 nM with vehicle treatment to 4516±502 nM with DNSP-11 treatment (t_(30)_ = 3.588, p = 0.0012). All data were analyzed using a two-tailed unpaired t-test *p<0.05. (B) Apomorphine (0.05 mg/kg) induced rotational behavior was assessed prior to infusion treatment (Pre) and once weekly for 4 weeks after DNSP-11 (100 µg) or vehicle treatment. Drug-induced rotational behavior is expressed as a percentage of vehicle treatment and showed a significant decrease in rotational behavior beginning one week after DNSP-11 treatment that lasted for all 4 weeks post DNSP-11. The data were analyzed using a one-way ANOVA for repeated measures (F_(4,39)_ = 4.807, p = 0.0005) with Bonferroni's multiple comparison test *p<0.05, **p<0.01, ***p<0.001. (C) DNSP-11 treatment significantly increased levels of DA, (74%) and DOPAC (132%) in the substantia nigra of unilateral 6-OHDA-lesioned rats. DA content was determined to be 34.7±6.4 ng/g in the vehicle treatment group and 59.1±7.3 ng/g in the DNSP-11 treatment group (t_(13)_ = 2.521, p = 0.0265). DOPAC tissue content was determined to be 7.10±1.40 ng/g in the vehicle treatment group and 16.48±4.01 ng/g (t_(13)_ = 2.33, p = 0.0364) in the DNSP-11 treatment group. All data were analyzed using a two-tailed unpaired t-test, * p<0.05.

Prior *in vivo* studies with GDNF have shown robust effects on both potassium- and amphetamine-evoked dopamine release 28 days after a single injection into the rat substantia nigra [27] indicating the functional effects of this trophic factor on dopamine signaling in the normal rat striatum. In our studies, 30 µg of DNSP-11 was injected into the right substantia nigra of normal young male Fischer 344 rats. Twenty-eight days after injection, *in vivo* microdialysis was performed in these animals to investigate dopamine neurochemistry in the ipsilateral striatum. Resting levels of dopamine, and the dopamine metabolites 3,4-dihydroxyphenylacetic acid (DOPAC) and homovanillic acid (HVA), were significantly increased by over 100% in the DNSP-11 treated rats as compared to controls (**Figure 4A**). These data support longer term effects of DNSP-11 on dopamine neuron function, and are analogous to prior results involving GDNF administration in rats and nonhuman primates [16], [28].

The *in vitro* studies and *in vivo* measures of the neurotrophic effects of DNSP-11 led us to investigate the potential neurorestorative properties of DNSP-11 to damaged dopamine neurons in a unilateral rat model of PD. Fischer 344 rats received dual-site unilateral injections of 6-OHDA to produce extensive destruction of the ascending dopaminergic system that resulted in a greater than 99% depletion of striatal dopamine content and a greater than 97% depletion of nigral dopamine content ipsilateral to the site of the 6-OHDA injections. Rats were tested 3–4 weeks after the injection of 6-OHDA using low-dose (0.05 mg/kg, i.p.) apomorphine to induce rotational behavior. In rats that rotated greater than 300 turns/ 60 minutes, 100 µg of DNSP-11 was injected into the substantia nigra ipsilateral to the 6-OHDA injections. DNSP-11 produced a significant ∼50% decrease in apomorphine-induced rotational behavior that was significant 1 week after administration and this effect was maintained for at least 4 weeks after DNSP-11 (**Figure 4B**). At 5 weeks, tissue samples of the substantia nigra and striatum from each rat were analyzed by high performance liquid chromatography coupled with electrochemical detection (HPLC-EC). A single injection of DNSP-11 was found to significantly increase levels of dopamine and the dopamine metabolite, DOPAC, by ∼100% in the substantia nigra, supporting that DNSP-11 has a powerful neurotrophic-like restorative effect on dopamine neurons in this animal model of late stage PD (**Figure 4C**). As observed with a single injection of GDNF [4], no significant changes in dopamine or its metabolites, DOPAC and HVA, were observed in the lesioned striatum (data not included).

**Figure 5:**
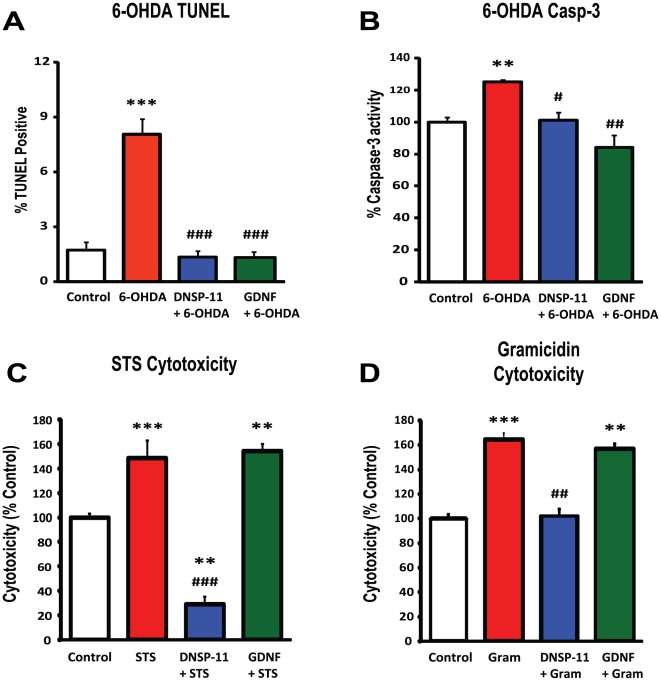
DNSP-11 functions differently than GDNF. Both DNSP-11 and GDNF protect against 6-OHDA toxicity as demonstrated by reductions in TUNEL staining at 24 h (A) and caspase-3 (B) activity at 3 h after 6-OHDA exposure. MN9D dopaminergic cells were incubated for 1 hour with either citrate buffer (control), 1 ng/mL of DNSP-11 or GDNF prior to 100 µM 6-OHDA exposure for 15 min. Data are + SD, one-way ANOVA with Tukey's post hoc analysis, *p<0.05, **p<0.01, ***p<0.001 vs. control; #p<0.05, ##p<0.01, ###p<0.001 vs. 6-OHDA. (C) DNSP-11 reduces staurosporine- induced cytotoxicity (1 µM) by ∼125% as measured by LIVE/DEAD® assay in dopaminergic B65 cells, whereas GDNF offers no protection at 20 hours after treatment. Control was citrate buffer alone; GDNF and DNSP-11 were added at 1 ng/mL. STS-staurosporine. (D) Similarly, DNSP-11 reduces gramicidin-induced cytotoxicity (1 µM) by ∼60%, whereas GDNF offers no protection at 20 hours after treatment. One-way ANOVA used to test for significance among groups, followed by Tukey's post hoc analysis (*p<0.05, **p<0.01, ***p<0.001 vs control; #p<0.05, ##p<0.01, ###p<0.001 vs toxin). Gram-gramicidin.

To evaluate DNSP-11's cellular neuroprotective properties, DNSP-11 was compared to GDNF in its protection against 6-OHDA-induced toxicity in the MN9D dopaminergic cell line. As seen in **Figures 5A & 5B**, 100 µM 6-OHDA significantly increased TUNEL staining and caspase-3 activity in MN9D cells. Pretreatment with DNSP-11 or GDNF produced a significant reduction in the percent of TUNEL positive cells and caspase-3 activity. To gain insight into DNSP-11's cellular mechanism, a DNSP-11 pull-down assay with cytosolic homogenate from isolated substantia nigra of normal young Fischer 344 rats was performed (Figure S1). Of the 16 proteins that were identified by MALDI-TOF mass spectrometry, 11 possess metabolic functions (**Table 2**) including glyceraldehyde-3-phosphate dehydrogenase (GAPDH) - a known neuroprotective drug target [29]–[32] with a link to PD [33] and apoptosis [34]. The prevalence of metabolic proteins is of note, given the mechanisms (and the emergence of genetic findings) suggesting a link between mitochondrial dysfunction and dopamine neuron loss in PD [35]–[39]. It is also noteworthy that none of these proteins are involved in the GDNF/GFRα1/RET signaling pathway.

**Table 2:** Identified cytosolic proteins pulled-down by DNSP-11 from substantia nigra of Fischer 344 rats.

Protein	Primary Function
*Aconitate Hydratase*	*Metabolism*
*Alpha Enolase*	*Metabolism*
*Aspartate Aminotransferase*	*Metabolism*
*Creatine Kinase*	*Metabolism*
*Fructose Bisphoshate Aldolase*	*Metabolism*
*Glutamate Dehydrogenase*	*Metabolism*
*Glutamine Synthetase*	*Metabolism*
*Glyceraldehyde 3 Phosphate Dehydrogenase*	*Metabolism/Apoptosis*
*L-Lactate Dehydrogenase B Chain*	*Metabolism*
*Malate Dehydrogenase*	*Metabolism*
*Pyruvate Kinase M1/M2*	*Metabolism*
*Heat Shock Cognate 71kDa Protein*	*Chaperone*
*Actin*	*Cytoskeleton*
*Tubulin*	*Cytoskeleton*
*Dihydropyrimidinase-Related Protein 2*	*Neuronal Development*
*Elongation Factor-1 Alpha 2*	*Translation*

Bound proteins were separated by 2D-PAGE (Figure S1), excised, trypsin digested and analyzed by MALDI-TOF MS/MS. MS data were compared to the Uniprot database [23] utilizing the Paragon™ algorithm [24] in ProteinPilot Version 2.0 (Applied Bioscience). The proteins listed were identified by ≥4 peptides with >99% confidence (ProteinPilot unused score ≥4).

**Figure 6:**
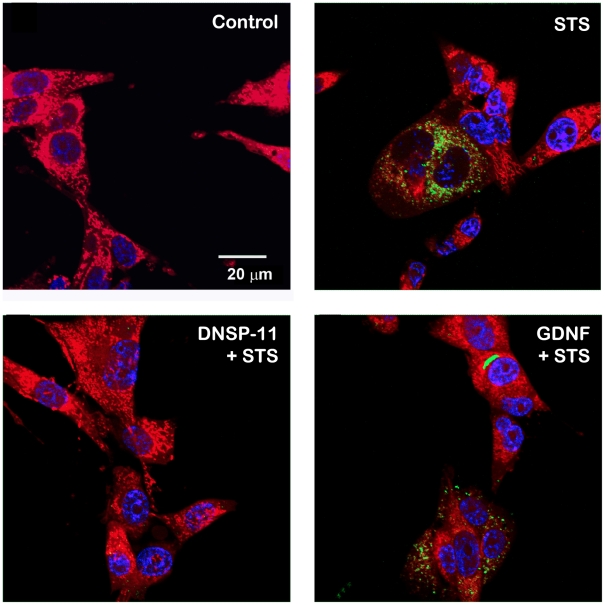
DNSP-11 prevents staurosporine-induced cytochrome c release from mitochondria. Confocal microscopy images demonstrate that DNSP-11 prevents cytochrome c (green) release from mitochondria (red) of B65 cells, supporting its protection from staurosporine-induced cytotoxicity (Figure 5C). GDNF does not prevent cytochrome c diffusion from the mitochondria when exposed to 1 µM staurosporine. The control was citrate buffer alone. The nucleus is stained blue in all images.

To further demonstrate the cellular mechanism differences between GDNF and DNSP-11, we examined DNSP-11's protective effects against staurosporine-induced apoptosis. Staurosporine, a nonselective protein kinase inhibitor and cytotoxin, promotes stress-induced apoptosis by triggering the release of cytochrome c from mitochondria and/or the loss of mitochondria potential [40]–[42]. In sympathetic and dopaminergic neurons, deprivation of GDNF was shown previously not to initiate the release of cytochrome c into the cytosol from the mitochondria, suggesting cell death occurs via a non-mitochondrial pathway [43], [44]. We found that DNSP-11 offers protection from staurosporine-induced and gramicidin-induced (a monovalent cation ionophore and mitochondria depolarization agent [41]) cytotoxicity in dopaminergic B65 cells, unlike GDNF, supporting that DNSP-11's neuroprotective effects involve mitochondria (**Figure 5C, 5D**). Staining of B65 cells showed that DNSP-11 protects against staurosporine-induced toxicity by preventing the release of cytochrome c from mitochondria (**Figure 6**). Furthermore, pull-down and ELISA analyses showed the absence of interaction between GFRα1 with DNSP-11 (Figure S2, S3). Collectively, these data support that DNSP-11 exhibits potent neurotrophic actions analogous to GDNF, but likely signals through pathways that do not directly involve the GFRα1 receptor.

## Discussion

Neurotrophic factors are a class of functionally related proteins that play a key role in neurite formation and growth during development and after injury [45]. Because of their native cellular function, neurotrophic factors have received considerable attention as potential therapeutic agents for neurodegenerative disorders, including PD. Perhaps the most promising neurotrophic factor for dopamine neurons has been GDNF. GDNF has been shown to increase the survival of cultured dopaminergic neurons [2], increase dopamine levels in the rat and monkey substantia nigra, and improve motor deficits with long-lasting effects in rats and nonhuman primate models of PD [3], [4], [25]. Two Phase I clinical trials support its beneficial effects in humans, but a failed Phase II trial and possible toxicity in an animal study have created controversy involving CNS treatment through the use of direct pump delivery [4]–[6].

Small molecules like DNSP-11 do not have the inherent pharmacological disadvantages and challenges associated with larger protein molecules. Furthermore, smaller peptides have the potential, with modification, to be administered orally or nasally, improving the potential for widespread use in the clinic [46]–[48]. DNSP-11, an 11 amino acid peptide possibly derived from the human GDNF prosequence, is a stable, easy to synthesize and purify molecule that opens up a new area of neurotrophic factor development. It shares many physiological and neurotrophic properties analogous to mature GDNF including: neuroprotection and promoting differentiation in primary dopamine neuron cell cultures; increasing dopamine release and metabolism *in vivo*; and decreasing apomorphine-induced rotations and enhancing dopamine function in the substantia nigra of 6-OHDA lesioned rats [7], [16], [49]. In addition, Immonen and colleagues have shown that the rat homolog of DNSP-11, called brain excitatory peptide (BEP), produces an increase in synaptic excitability in rat CA1 pyramidal neurons through possible involvement of a G-protein coupled receptor [15]. Collectively these data support that the pro-peptides of GDNF, and perhaps other homologous neurotrophic factor family members, such as neurturin and artemin, may have novel, long-overlooked physiological and potentially therapeutic functions.

Recent evidence suggests that functional peptide sequences are present in unexpected sources. For neurotrophic proteins such as GDNF, it is initially produced *in vivo* as an immature precursor that is processed proteolytically by furin-type endopeptidases, to liberate the mature functional protein from the propeptide [50]. While the function of the mature growth factors has been long established, their prosequences have been thought only to be involved in assisting protein folding and secretion [10], [11]. However, the high degree of sequence conservation between prodomains amongst different species challenges this dogma and strongly suggests additional biological functions [9]. Recent evidence suggests that functional peptide sequences exist in the prodomains of the neurotrophic factors NGF and BDNF [12]–[14], [50]–[52]. Here we have described an 11-mer peptide predicted to be derived from proGDNF that mimics many of GDNF's neurotrophic actions, but is not a ligand for the GFRα1 receptor. Additional studies are needed to investigate this potential new class of signalling peptides, by focusing on potential metabolic/mitochondrial mechanisms and the striatal delivery of DNSP-11 (analogous to GDNF), which may have beneficial effects in the clinical treatment of neurodegenerative diseases such as PD.

## Supporting Information

Figure S1 2D PAGE analysis of the binding partners of DNSP-11. In the presence (top) and absence (bottom) of bDNSP-11 with streptavidin magnetic beads. F344 substantia nigra was homogenized in homogenization buffer and cytosolic fraction (supernatant) collected after 30 minutes at 100,000 g. 50 µg of bDNSP-11 was incubated with fraction for 15 minutes on ice. Sample was added to streptavidin magnetic beads, pelleted, and washed four times in homogenization buffer. Bound proteins were eluted by Solubilization/Rehydration Solution (7 M Urea, 2 M Thiourea, 50 mM DTT, 4% CHAPS, 1% NP-40, 0.2% Carrier ampholytes, 0.0002% Bromophenol blue), and analyzed by 2D-PAGE and later identified by MALDI-TOF MS/MS (Table 1).
(0.41 MB DOC)

Figure S2 In vitro pull down assay determines that DNSP-11 does not bind to the GFRα1 receptor. A solution of 25 µL GFRα1 (1 mg/mL) was incubated with 50 µL of Dynabeads® (Invitrogen) in wash and bind buffer (0.1 M sodium phosphate, pH 8.2, 0.01% Tween® 20) for 10 minutes at room temperature. The beads were then washed three times in 100 µL of wash and bind buffer. 2 µg of GDNF was added and incubated for 1 hour at 4°C. 25 µL GFRα1 (1 mg/mL) was incubated with 40 µg of biotinylated DNSP-11 (bDNSP-11) for 1 hour at 4°C. They were then added to 50 µL of hydrophilic streptavidin magnetic beads (New England Biolabs) and incubated for an hour at 4°C. Expected binding was observed between GDNF and GFRα1. However, no binding was observed between bDNSP-11 and GFRα1. F-Flow through, E-Elution.
(0.07 MB DOC)

Figure S3 Direct ELISA binding assay to verify DNSP-11 does not interact with the GFRα1 receptor. A microtiter plate was coated with 500 ng/ml of either GDNF or DNSP-11 in 50 mM carbonate buffer (pH 9.6) overnight at 4°C, washed three times with PBS plus 0.05% Tween-20 (PBST) and blocked with 2% BSA in PBS (blocking buffer) at 37°C for 1 hour. Then, GFRα1/Fc receptor (R&D Systems) was added at a serial dilution (range 0–2 µg/ml) in blocking buffer. After 2 hours of incubation at room temperature, the wells were washed three times in PBST and then incubated with goat anti-human IgG (Fc specific) (1∶10,000, Sigma) in blocking buffer for 2 hours. Following three washes with PBST and incubation with peroxidase-conjugated horse anti-goat IgG (1∶10,000, Vector lab) in blocking buffer for 1 hour, wells were washed three times in PBST and two times in dH_2_O. The reaction was developed with 3,3′,5,5′-tetramethyl benzidine (TMB) substrate (Bio-Rad) for 10 minutes and stopped by addition of 1 N HCl. For each sample, absorbance values were recorded at 450 nm in duplicate. The wells without GFRα1/Fc receptor were used as control. Significant binding was only observed with GDNF. No binding above background was observed with DNSP-11.
(0.06 MB DOC)
