# Supplementary figures and images for: Dopamine Neuron Stimulating Actions of a GDNF Propeptide
Source: PLoS One. 2010 Mar 18;5(3):e9752. doi: 10.1371/journal.pone.0009752 (PMC2841203; doi:10.1371/journal.pone.0009752)

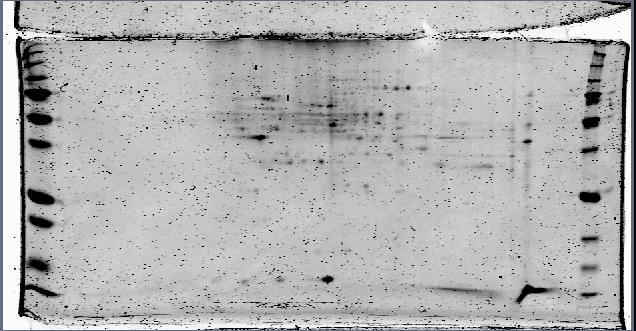


**
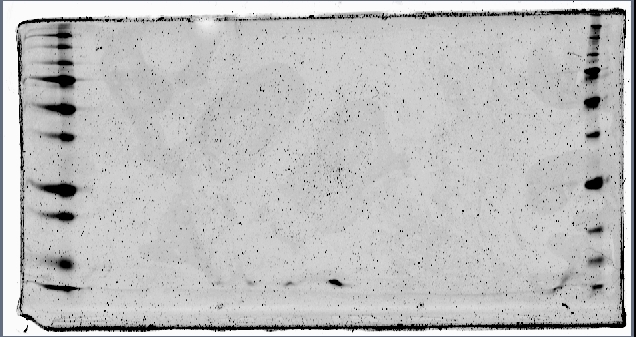
**

**FIGURE S1**

Supplement: Figure S1 — 2D PAGE analysis of the binding partners of DNSP-11. In the presence (top) and absence (bottom) of bDNSP-11 with streptavidin magnetic beads. F344 substantia nigra was homogenized in homogenization buffer and cytosolic fraction (supernatant) collected after 30 minutes at 100,000 g. 50 µg of bDNSP-11 was incubated with fraction for 15 minutes on ice. Sample was added to streptavidin magnetic beads, pelleted, and washed four times in homogenization buffer. Bound proteins were eluted by Solubilization/Rehydration Solution (7 M Urea, 2 M Thiourea, 50 mM DTT, 4% CHAPS, 1% NP-40, 0.2% Carrier ampholytes, 0.0002% Bromophenol blue), and analyzed by 2D-PAGE and later identified by MALDI-TOF MS/MS (Table 1). (0.41 MB DOC) [file pone.0009752.s001.doc]
