# Supplementary material for: Dopamine Neuron Stimulating Actions of a GDNF Propeptide
Source: PLoS One. 2010 Mar 18;5(3):e9752. doi: 10.1371/journal.pone.0009752 (PMC2841203; doi:10.1371/journal.pone.0009752)
Supplement: Figure S2 — In vitro pull down assay determines that DNSP-11 does not bind to the GFRα1 receptor. A solution of 25 µL GFRα1 (1 mg/mL) was incubated with 50 µL of Dynabeads® (Invitrogen) in wash and bind buffer (0.1 M sodium phosphate, pH 8.2, 0.01% Tween® 20) for 10 minutes at room temperature. The beads were then washed three times in 100 µL of wash and bind buffer. 2 µg of GDNF was added and incubated for 1 hour at 4°C. 25 µL GFRα1 (1 mg/mL) was incubated with 40 µg of biotinylated DNSP-11 (bDNSP-11) for 1 hour at 4°C. They were then added to 50 µL of hydrophilic streptavidin magnetic beads (New England Biolabs) and incubated for an hour at 4°C. Expected binding was observed between GDNF and GFRα1. However, no binding was observed between bDNSP-11 and GFRα1. F-Flow through, E-Elution. (0.07 MB DOC) [file pone.0009752.s002.doc]

**FIGURE S2**
