# Supplementary material for: Dopamine Neuron Stimulating Actions of a GDNF Propeptide
Source: PLoS One. 2010 Mar 18;5(3):e9752. doi: 10.1371/journal.pone.0009752 (PMC2841203; doi:10.1371/journal.pone.0009752)
Supplement: Figure S3 — Direct ELISA binding assay to verify DNSP-11 does not interact with the GFRα1 receptor. A microtiter plate was coated with 500 ng/ml of either GDNF or DNSP-11 in 50 mM carbonate buffer (pH 9.6) overnight at 4°C, washed three times with PBS plus 0.05% Tween-20 (PBST) and blocked with 2% BSA in PBS (blocking buffer) at 37°C for 1 hour. Then, GFRα1/Fc receptor (R&D Systems) was added at a serial dilution (range 0–2 µg/ml) in blocking buffer. After 2 hours of incubation at room temperature, the wells were washed three times in PBST and then incubated with goat anti-human IgG (Fc specific) (1∶10,000, Sigma) in blocking buffer for 2 hours. Following three washes with PBST and incubation with peroxidase-conjugated horse anti-goat IgG (1∶10,000, Vector lab) in blocking buffer for 1 hour, wells were washed three times in PBST and two times in dH2O. The reaction was developed with 3,3′,5,5′-tetramethyl benzidine (TMB) substrate (Bio-Rad) for 10 minutes and stopped by addition of 1 N HCl. For each sample, absorbance values were recorded at 450 nm in duplicate. The wells without GFRα1/Fc receptor were used as control. Significant binding was only observed with GDNF. No binding above background was observed with DNSP-11. (0.06 MB DOC) [file pone.0009752.s003.doc]

**FIGURE S3**
